# Supplementary material for: Sideritis scardica extracts inhibit aggregation and toxicity of amyloid-β in Caenorhabditis elegans used as a model for Alzheimer’s disease
Source: PeerJ. 2018 Apr 30;6:e4683. doi: 10.7717/peerj.4683 (PMC5933321; doi:10.7717/peerj.4683)
Supplement: Figure S1 [file peerj-06-4683-s001.pptx]

## Slide 1
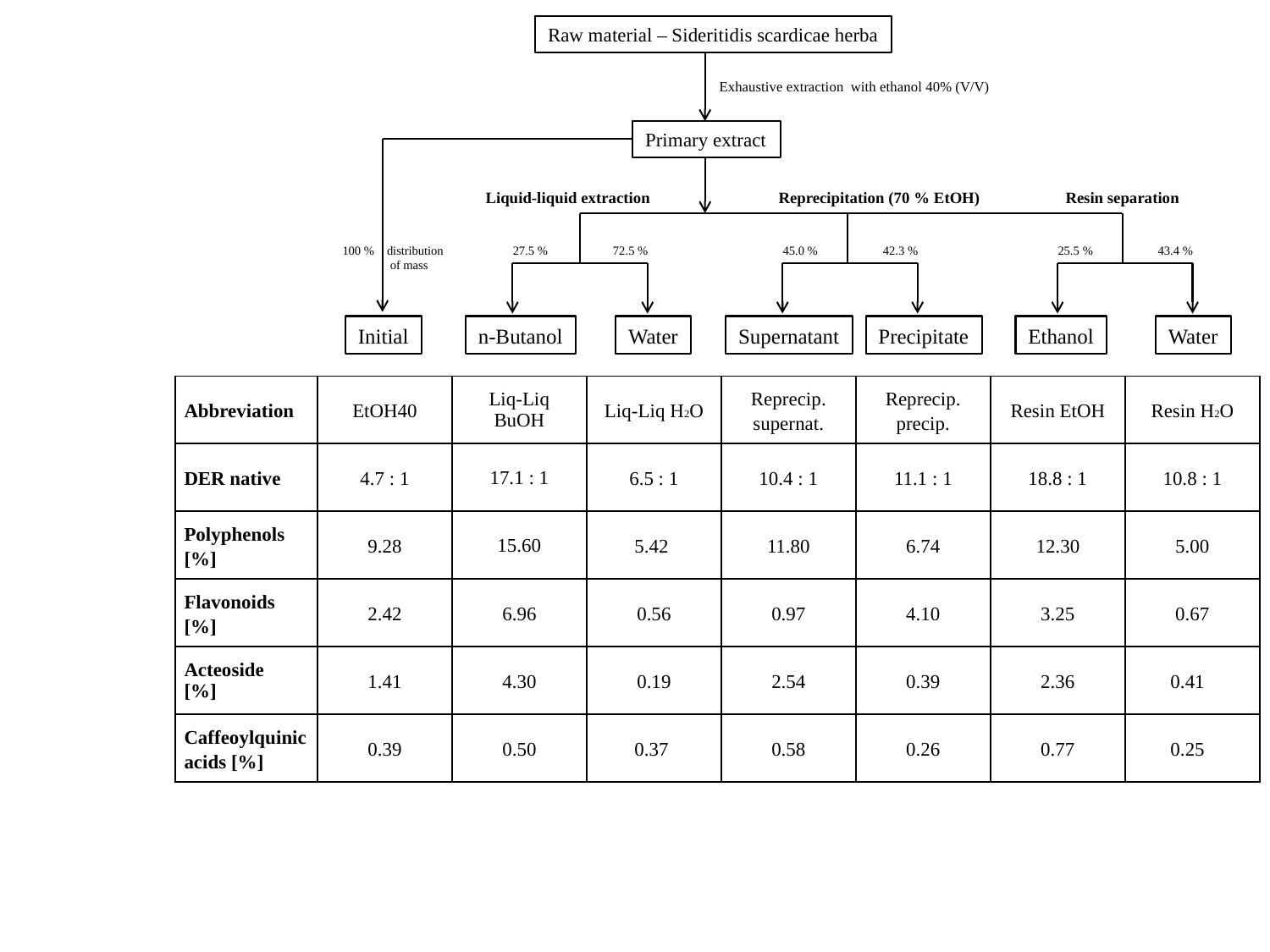

Raw material – Sideritidis scardicae herba
Exhaustive extraction with ethanol 40% (V/V)
Primary extract
Liquid-liquid extraction
Reprecipitation (70 % EtOH)
Resin separation
100 % distribution
 of mass
27.5 %
72.5 %
45.0 %
42.3 %
25.5 %
43.4 %
Initial
n-Butanol
Water
Supernatant
Precipitate
Ethanol
Water
| Abbreviation | EtOH40 | Liq-Liq BuOH | Liq-Liq H2O | Reprecip. supernat. | Reprecip. precip. | Resin EtOH | Resin H2O |
| --- | --- | --- | --- | --- | --- | --- | --- |
| DER native | 4.7 : 1 | 17.1 : 1 | 6.5 : 1 | 10.4 : 1 | 11.1 : 1 | 18.8 : 1 | 10.8 : 1 |
| Polyphenols [%] | 9.28 | 15.60 | 5.42 | 11.80 | 6.74 | 12.30 | 5.00 |
| Flavonoids [%] | 2.42 | 6.96 | 0.56 | 0.97 | 4.10 | 3.25 | 0.67 |
| Acteoside [%] | 1.41 | 4.30 | 0.19 | 2.54 | 0.39 | 2.36 | 0.41 |
| Caffeoylquinic acids [%] | 0.39 | 0.50 | 0.37 | 0.58 | 0.26 | 0.77 | 0.25 |
