# Supplementary material for: Sideritis scardica extracts inhibit aggregation and toxicity of amyloid-β in Caenorhabditis elegans used as a model for Alzheimer’s disease
Source: PeerJ. 2018 Apr 30;6:e4683. doi: 10.7717/peerj.4683 (PMC5933321; doi:10.7717/peerj.4683)
Supplement: Supplemental Information 1 — The worms were treated with the highest used concentrations of the extracts and fractions. A reduced body length would point to toxic effects to the worms or antimicrobial properties against the food source of the worms (E. coli). All concentrations were deemed suitable [file peerj-06-4683-s002.docx]

| **Substance** | **Concentration [µg/mL]** | **Body length [mm]** | **SEM [mm]** | **Significance** |
| --- | --- | --- | --- | --- |
| Untreated | N/A | 1149.9 | 30.0 | N/A |
| H_2_O | 600 | 1138.8 | 21.0 | n.s. |
| EtOH20 | 1000 | 1136.6 | 38.9 |  |
| EtOH40 | 600 | 1180.5 | 69.2 |  |
| EtOH50 | 600 | 1161.6 | 33.7 |  |
| EtOH70 | 600 | 1197.2 | 50.8 |  |
| EGCG | 100 | 1172.6 | 6.0 |  |
| Liq-Liq BuOH | 400 | 1175.7 | 32.4 |  |
| Liq-Liq H_2_O | 400 | 1169.9 | 54.6 |  |
| Reprecip. supernat. | 400 | 1151.1 | 32.6 |  |
| Reprecip. precip. | 400 | 1162.0 | 32.7 |  |
| Resin EtOH | 400 | 1131.6 | 18.9 |  |
| Resin H_2_O | 400 | 1113.5 | 21.0 |  |

n.s. not significant

N/A not applicable

Growth of the worms is not inhibited by highest used concentrations. The substances are thus deemed non-toxic or negative for *C. elegans*.
