# Supplementary material for: Sideritis scardica extracts inhibit aggregation and toxicity of amyloid-β in Caenorhabditis elegans used as a model for Alzheimer’s disease
Source: PeerJ. 2018 Apr 30;6:e4683. doi: 10.7717/peerj.4683 (PMC5933321; doi:10.7717/peerj.4683)
Supplement: Supplemental Information 2 [file peerj-06-4683-s005.pdf]

Sideritis scardica GRISEB.

Verfasser

F. Heiner, B. Feistel, J. Reichling

Übersicht

S [1] > Sideritis [2] > **Sideritis scardica GRISEB.** [3]

Gliederung

G Sideritis [4]
A **Sideritis scardica GRISEB.** [5]
D Sideritidis scardicae herba [11] [6], [20] [7], [45] [8]

Systematik

Synonyme: *Navicularia scardica* (GRISEB.) SOJAK; *Sideritis florida* BOISS. & HELDR. Die früheren Synonyme *Sideritis raeseri* ssp. *florida* (BOISS. & HELDR.) PAPAN. & KOKKINI und *Sideritis scardica* ssp. *longibracteata* PAPAN. PAPAN. & KOKKINI haben nach heutiger Einschätzung praktisch keine Bestätigung erfahren und werden kaum genutzt [5] [9], [8] [10], [21] [11], [22] [12], [63] [13].

Sonstige Bezeichnungen: Greek mountain tea, Mountain tea, Shepherd's tea, Ironwort, Olympos tea; deutsch: Griechischer Bergtee, Griechisches Eisenkraut; öster.::Püringertee; alban.:Caj Mali (Bergtee);; bulgar.:Mursalski Tee, Pirinski Tee, Pirin-Tee; griech.:Tsai tou vounou (Bergtee); mazedon.: Planinski Tee [21] [11], [23] [14].

Botanische Beschreibung: Mehrjähriger, verzweigter Halbstrauch (Abb. 1), der mit einem kräftigen Wurzelstock (Abb. 2) ausgestattet ist. Stängel an der Basis verzweigt und verholzt, die oberen Stängelteile sind krautig, vierkantig und 15 bis 40 cm hoch. Die Stängel sind bis zur Infloreszenz wollig behaart. Die Grundblätter sind grau-filzig behaart, kreuzgegenständig, linealisch bis schmal lanzettlich. Die mittleren und oberen Stängelblätter sind meist sitzend, behaart und mit Achselknospen versehen. Die Blattspreite ist länglich-elliptisch oder lanzettlich-linear mit gekerbtem Blattrand. Der ährenförmige Blütenstand (Infloreszenz) ist ca. 4 bis 9 cm lang und besteht aus 8 bis 17 Scheinquirlen (Abb. 1). Die Deckblätter (Brakteen) sind ganzrandig, eiförmig, 10 bis 20 mm lang und spitz zulaufend. Sie sind ein- bis zweimal so lang wie die Blüten, mit Drüsen bedeckt und von grünlich bis gelber Farbe. In den Achseln der Deckblätter sitzen zitronengelbe, zygomorphe Blüten in Form von Scheinquirlen. Fruchtform: Klausenfrüchte. Blütezeit: Juli bis August [24] [15] - [26] [16].

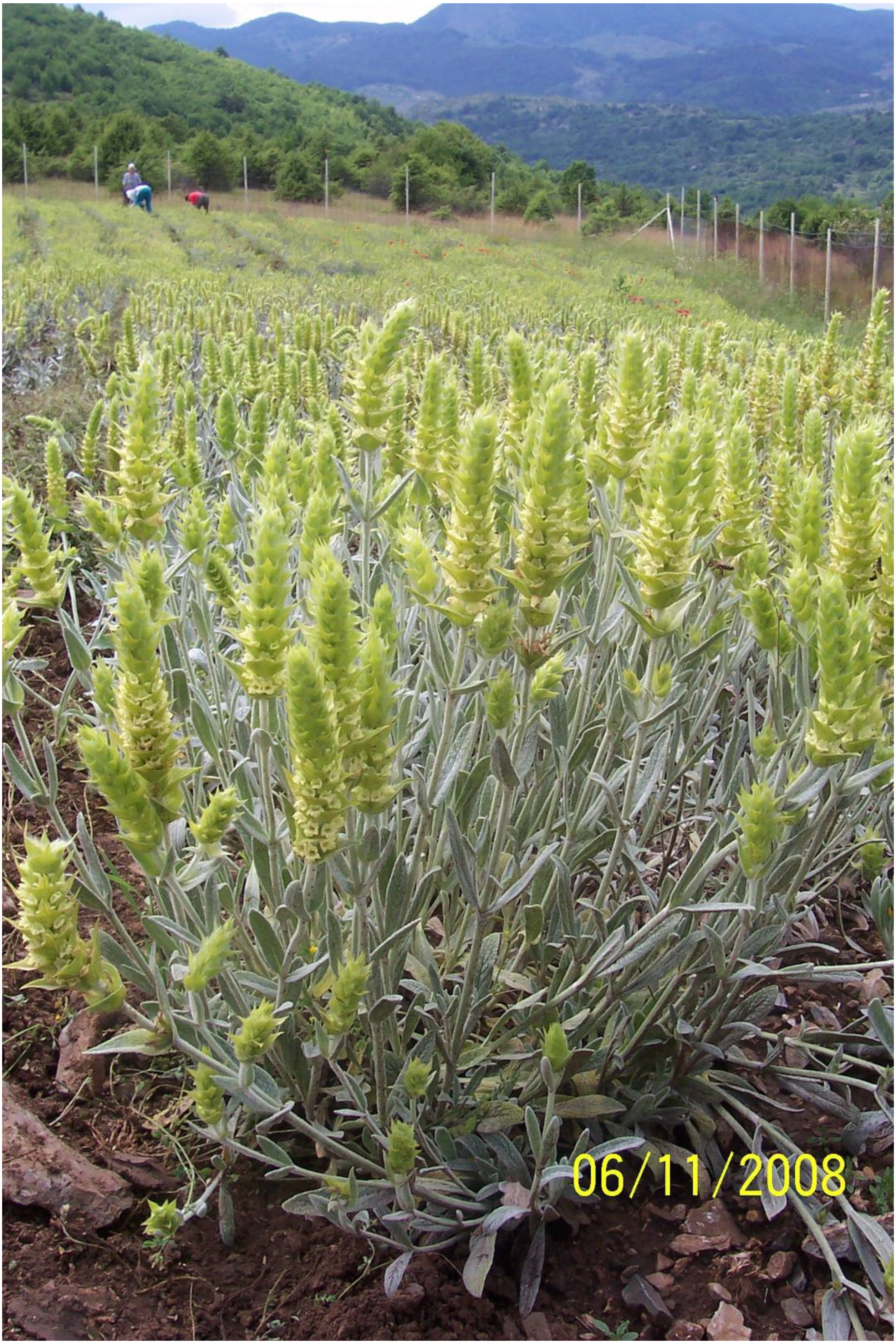
06/11/2008

Sideritis scardica (Griechischer Bergtee), oberirdische Teile der Pflanze mit Blütenstand

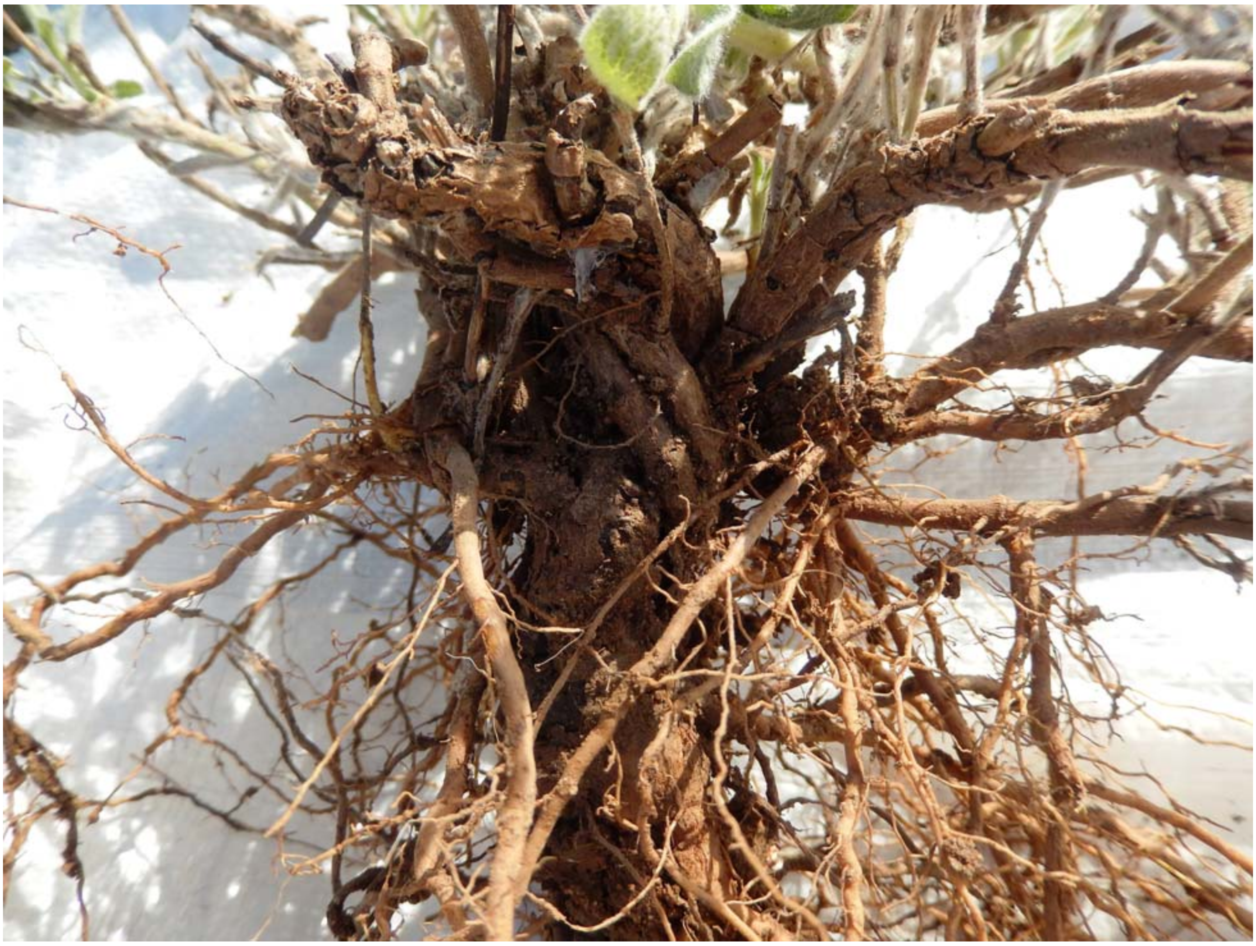

Sideritis scardica, unterirdische Teile der Pflanze, Wurzelstock mit Wurzeln

Verbreitung

Sideritis scardica ist endemisch auf der Balkan-Halbinsel, auffindbar in Süd-West Albanien, Mazedonien, Bulgarien, Serbien, Griechenland und der Türkei. Der Griechische Bergtee wächst in alpinen und subalpinen Vegetationsgürteln, vor allem auf offenen, steinigen (kalkhaltig und durchlässig), trockenen Hängen, die lange Zeit der Sonne ausgesetzt sind. Obschon die Pflanze sowohl kälte- (-20 °C) als auch hitzetolerant (+40 °C) ist, wächst sie wild nur in diesem, hauptsächlich mediterranen Gebiet [15] [17], [20] [7], [21] [11], [27] [18] - [30] [19].

Anbau/Haltung

Ein Großteil der im Handel befindlichen Droge stammt nach wie vor aus Wildsammlungen. Inzwischen wird die Pflanze u. a. in Albanien, Bulgarien, Griechenland, Mazedonien und der Türkei angebaut [15] [17], [23] [14], [31] [20]. In Bulgarien werden regionenspezifisch verschiedene Kultivare angebaut (Abb. 3) [25] [21]. Die Ernte erfolgt traditionell händisch durch Schneiden des Krautes; ebenso die Trocknung in Bündeln an der warmen Luft.

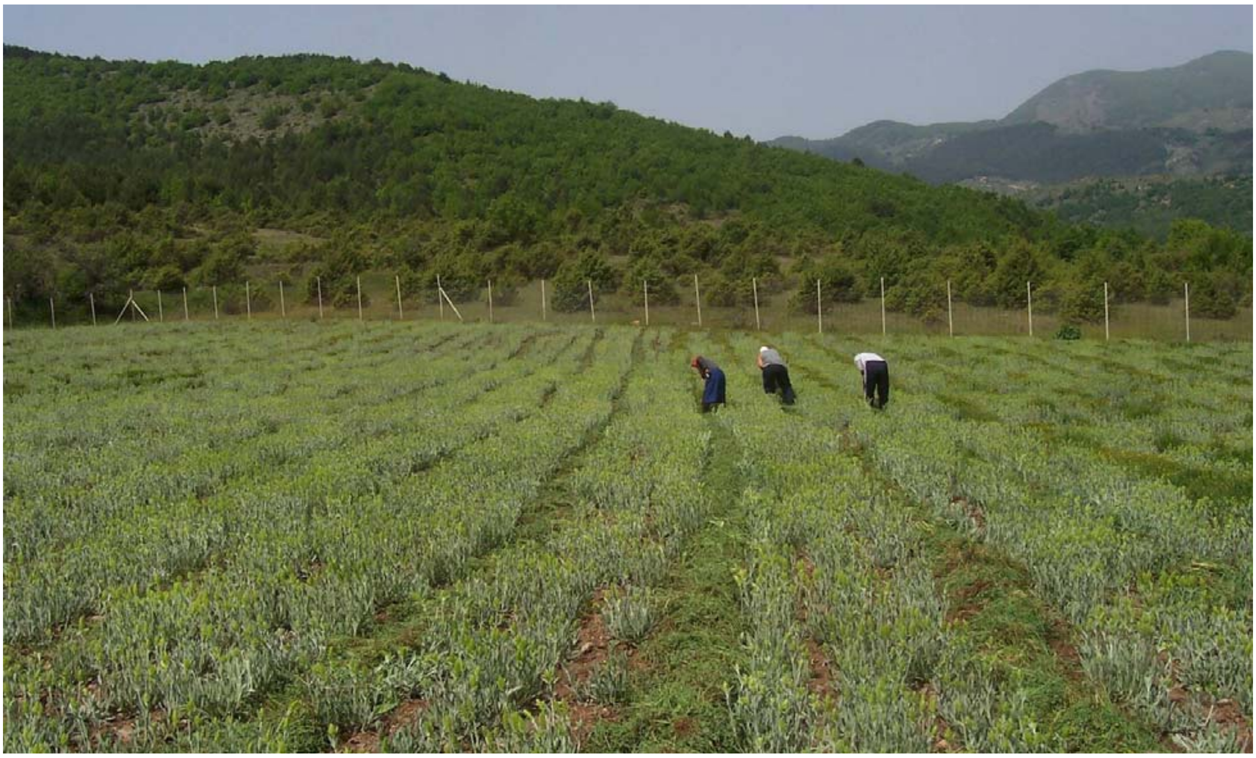

Kommerzieller Anbau von *Sideritis scardica* in Bulgarien, Martin Bauer GmbH

Inhaltsstoffe

Die oberirdischen Teile der Pflanze weisen einen relativ hohen Gehalt an Polyphenolen auf (ca. 5 %). Während der Knospenbildung wurde der höchste Gehalt an Flavonoiden (ca. 0,34 %) und zu Beginn der Vegetationsperiode der höchste Gehalt an (Lamiaceen-) Gerbstoffen (bis 7,93 %) gemessen [32] [22]. Neben Flavonoiden und Tanninen liegen in der Pflanze noch Phenylethanoid-Glykoside und Hydroxyzimtsäure-Derivate vor [32] [22], [33] [23]. Im Vergleich zu anderen Lamiaceen enthalten die oberirdischen Teile der Pflanze relativ geringe Konzentration an ätherischen Ölen (ca. 0,03 %). GC/MS-Analysen zeigten das Vorhandensein von Monoterpenen, Sesquiterpenen, Diterpenen, aliphatischen und aromatischen Substanzen sowie Fettsäuren (z. B. Palmitinsäure) und Oleylalkohol [11] [24], [33] [23] - [42] [25]. Die Zusammensetzung des ätherischen Öls ist sehr abhängig vom Fundort und der Jahreszeit [15] [17]. Darüber hinaus sind in den Blättern Diterpene, hauptsächlich vom ent-Kauren-Typ sowie Mineralstoffe (Na, K, Ca, Mg, Fe, Zn, Mn im ppm-Bereich) vorhanden [44] [26]. Auch toxische Inhaltsstoffe wie z. B. Blei, Cadmium und Arsen können in Spuren vorkommen [33] [23], [44] [26], welche jedoch in den traditionellen Zubereitungen wie Tee oder Tinkturen nicht nachweisbar sind.

Drogen

Sideritidis scardicae herba [11] [6], [20] [7], [45] [8].

Literatur

1. Scheen AC, Bendiksby, M, Ryding O et al. (2010) Molecular phylogenetics, character evolution, and suprageneric classification of Lamioideae (Lamiaceae). Ann Missouri Bot Gard 97:191 217
2. Salmaki Y, Zarre S, Ryding O et al. (2013) Molecular phylogeny of tribe Stachydeae (Lamiaceae subfamily Lamioideae). Mol Phylogenet Evol 69:535 551
3. Huynh KL (1972) Le pollen et la systématique du genre Sideritis L. (Labiatae). Bull Museum Natl Hist Nat (Paris) 45:1 28
4. Huber-Morath A (1982) Sideritis. In: Peter Hadland Davis (Eds.): Flora of Turkey and the East Aegean Islands. Vol. 7 (Orobanchaceae to Rubiaceae). Edinburgh University Press, S. 178 199
5. Papanikolaou K, Kokkini S (1982) A taxonomic revision of Sideritis L. section Empedoclia (Rafin.) Benntham (Labiatae) in Greece. In: Margaris, N., Koedam, A., Vokou, D. (Eds.), Aromatic Plants: Basic and Applied Aspects. Artinus Nijhov Publ., The Hague, S. 101 128
6. Pérez de Paz P, Negrín-Sosa L (1992) Revisión taxonómica de Sideritis L. Subgénero Marrubiastrum (Moench) Mend.-Heur. Phanerogamarum Monographiae, Tomus XX. J. Cramer, Berlin
7. Obón de Castro C, Rivera-Núñez D (1994) A taxonomic revision of the section Sideritis (genus Sideritis) (Labiatae). Phanerogamarum Monographiae Tomus XXI. J. Cramer, Berlin
8. Cantino PD, Abu-Asab MS (1994) Systematic implications of pollen morphology in subfamilies Lamioideae and Pogostemonoideae (Labiatae). Ann Missouri Bot Gard 81:653 686
9. Morales R (2010) Sideritis. In: Castroviejo S, Morales R, Quintana A, et al. (Eds.), Flora Iberica XII. Plantas vasculares de la Península Ibérica e Islas Baleares. Real Jardín Botánico, CSIC, Madrid, S 1 56
10. Bendiksby M, Lisbeth T, Scheen AC et al. (2011) An updated phylogeny and classification of Lamiaceae subfamily Lamioideae. Taxon 60:471 484
11. González-Burgos E, Carretero ME, Gómez-Serranillo MP (2011) Sideritis spp.: uses, chemical composition and pharmacological activities - a review. J Ethno pharmacol 135:209 225
12. Güvenç A, Duman H (2010) Morphological and anatomical studies of annual taxa of Sideritis L. (Lamiaceae), with notes on chorology in Turkey. Turk J Bot 34:83 104
13. Davis PH (1982) Flora of Turkey and the East Aegean Islands. Edinburgh University Press, Edinburgh, S. 7
14. Davis PH, Mill RR, Kit Tan (1988) Flora of Turkey and the East Aegean Islands. Edinburgh University Press, Edinburgh, S. 10
15. Todorova M, Trendafilova A (2014) *Sideritis scardica* Griseb., an endemic species of Balkan peninsula: Traditional uses, cultivation, chemical composition, biologicalactivity. Review. J Ethnopharmacol 152:256 265
16. Fraga BM (2012) Phytochemistry and chemotaxonomy of Sideritis species from the Mediterranean region. Phytochem 76:7 24
17. Petreska J, Stefkov G, Kulevanova S et al. (2011a) Phenolic compounds of mountain tea from the Balkans: LC/DAD/ESI/MSn profile and content. Nat Prod Commun 6:21 30
18. Petreska J, Stefova M, Ferreres F et al. (2011b) Potential bioactive phenolics of Macedonian Sideritis species used for medicinal "Mountain Tea". Food Chem 125:13 20
19. Bojovic D, Jankovic S, Potpara Z et al. (2011) Summary of the phytochemical research performed to date on Sideritis species. Ser J Exp Clin Res 12:109 122
20. Heywood V (1972) Sideritis L. In: Tutin T, Heywood V, Burges N et al. (Eds.), Flora Europaea, 3. Cambridge University Press, Cambridge, S. 134 138
21. Lim TK (2014) Edible Medicinal and Non Medicinal Plants. Volume 8, Flowers. Dordrecht: Springer Science + Business Media
22. Halácsy E (1902) Conspectus Florae Graecae. G.Engelmann, Lipsiae, Vol. II, 497–502
23. Feistel B (2013) Griechisches Eisenkraut - Mental fit mit Griechischem Bergtee. Vitalstoffe 3:34–37

24. Assenov I, Ganchev G (1978) Morphological characteristics of *Sideritis scardica* Griseb. Pharmacia 2:29–32

25. Evstatieva L, Bakalova K, Protich N (1990) Ecological and biological peculiarities of *Sideritis scardica* Griseb. Plant Sci. 27:77–80 (bulgar.)

26. Evstatieva L (2011) *Sideritis scardica* Griseb. In: Peev D (Ed.), Red Data Book of the Republic of Bulgaria, Joint Edition of the Bulgarian Academy of Sciences & Ministry of Environment and Water of Bulgaria, Digital Edition, Vol. 1, http:// e-ecodb.bas.bg/rdb/en/

27. Baden C (1991) Sideritis L. In: Strid A, Tan K (Eds.), The Mountain Flora of Greece, 2. Edinburgh University Press, Edinburgh, S. 84–91

28. Duman H (2000) Sideritis L. In: Güner A, Ozhatay N, Ekim T, Basher KHC (Eds.), Flora of Turkey and the East Aegean Islands, 11. Edinburgh University Press, Edinburgh, S. 201–204

29. Yordanova M, Apostolova I (2000) Estimation of the status of representative populations of *Sideritis scardica* Griseb. in the Rhodopi Mts. Phytol Balc 6:4357

30. Petrova A, Vladimirov V (2009) Red list of Bulgarian vascular plants. Phytol Balc 15:63–94

31. Skrubis G (1984) The Greek mountain tea. In: Spice, medicinals, aromatics. Department of aromatic plants, agriculture research center of northern Greece Thessaloniki - Greece. Acta Horticultura 144:25–29

32. Evstatieva L (2002) Variation in the flavonoids and tannins content of S. scardica Griseb. Annual of Sofia University "Kliment Ohridski" 90. Faculty of Biology, Sofia, S. 99–105

33. Karapandzova M, Qazimi B, Stefkov G et al. (2013) Chemical characterization, mineral content and radical scavenging activity of *Sideritis scardica* and *S. raeseri* from R. Macedonia and R. Albania. Nat Prod Commun 8:639–644

34. Kokkalou E (1987) Constituents entrainables à la vapeur d'eau de *Sideritis scardica* Griseb subsp. scardica. Plant Med Phytother 21:262–266

35. Baser KHC, Kirimer N, Tümen G (1997) Essential oil of *Sideritis scardica* Griseb subsp. scardica. J Essent Oil Res 9:205–207

36. Kostadinova E, Nikolova D, Alipieva K et al. (2007) Chemical constituents of the essential oils of *Sideritis scardica* Griseb. and *Sideritis raeseri* Boiss and Heldr. from Bulgaria and Macedonia. Nat Prod Res 21:319–

37. Todorova M, Christov R, Evstatieva L (2000) Essential oil composition of three Sideritis species from Bulgaria. J Essent Oil Res 12:418–420

38. Trendafilova AB, Todorova MN, Evstatieva LN et al. (2013) Variability in the essential-oil composition of *Sideritis scardica* Griseb. from native bulgarian populations. Chem Biodivers 10:484–492

39. Qazimi B, Karapandzova M, Stefkov G et al. (2010) Chemical composition of ultrasonic assisted n-hexane extracts of *Sideritis scardica* Grieseb. and *Sideritis raeseri* Boiss. & Heldr. (Lamiaceae) from Macedonia and Albania. Maced. Pharmaceut Bull 56:45–56

40. Kostadinova E, Alipieva K, Stefova M et al. (2008) Influence of cultivation on the chemical composition and antimicrobial activity of Sideritis spp. Pharmacogn Mag 4:102 –106

41. Tadić V, Bojović D, Arsić I et al. (2012a) Chemical and antimicrobial evaluation of supercritical and conventional *Sideritis scardica* Griseb. (Lamiaceae) extracts. Molecules 17:2683–2703

42. Taskova R, Mitova M, Anchev M et al. (1997) Iridoids, flavonoids and terpenoids as taxonomic markers in Lamiaceae, Scrophulariaceae and Rubiaceae. Bocconea 5:631–636

43. Venturella P, Bellino A (1979) Isolation and partial synthesis of ent-18-acetoxykaur-16-ene-3β,7α,15β-triol from *Sideritis scardica*. Phytochem 1:1571–1572

44. Žarković B, Djordjević S, Blagojević S (1993) Comparison of the mineral composition of the plant *Sideritis scardica* Griseb., Lamiaceae and corresponding soil substrate. Arh Farm 43:233–237

45. Nikolov S (2006) Encyclopedia of the Medicinal Plants in Bulgaria. Publishing House "Trud", Sofia, S. 347–348. (bulgar.)

46. Bojchinov A (1943) Pharmacognostic study of *Sideritis scardica* Griseb. Aptek Pregled 9:151–158 (bulgar.)

47. Koleva I, Linssen JPH, van Beek TA et al. (2003) Sideritis species (Labiatae) grown in Bulgaria. J Sci Food Agric 83:809–819

48. Tadić VM, Djordjević S, Arsić I et al. (2007) Anti-inflammatory and antimicrobial activity of *Sideritis scardica* extracts. Planta Med 73:P-098

49. Proestos C, Zoumpoulakis P, Sinanoglou VJ (2013) Determination of plant bioactive compounds. Antioxidant capacity and antimicrobial screening. FMFI 2:26–

50. Mihaylova DSP, Anna L, Albert KI (2014) A study on the antioxidant and antimicrobial activities of pressurized-liquid extracts of *Clinopodium vulgare* and *Sideritis scardica*. Agro Food Ind Hi Tech 25:55–58

51. Tadić VM, Djordjević S, Arsić I et al. (2008) Cytotoxic activity and antioxidative properties of *Sideritis scardica* extracts, Planta Med 74:PA206

52. Tadić VM, Jeremic I, Dobric S et al. (2012) Anti-inflammatory, gastroprotective, and cytotoxic effects of *Sideritis scardica* extracts. Planta Med 78:415–427

53. Ivanova D, Gerova D, Chervenkov T et al. (2005) Polyphenols and antioxidant capacity of Bulgarian medicinal plants. J Ethnopharmacol 96:145–150

54. Danesi F, Saha S, Kroon PA et al. (2013) Bioactive-rich *Sideritis scardica* tea (mountain tea) is as potent as *Camellia sinensis* tea at inducing cellular antioxidant defences and preventing oxidative stress. J Sci Food Agric 93: 3558–3564

55. Tadić VM, Marković G, Jeremic et al. (2009) Antiglioma action of *Sideritis scardica* extracts, Planta Med 75:PE67 (DOI: 10.1055/s-0029-1234628).

56. Jeremić I, Tadić V, Isaković A et al. (2013) The mechanisms of in vitro cytotoxicity of mountain tea, *Sideritis scardica*, against the C6 glioma cellline. Planta Med 79:1516 –1524

57. Knörle R (2012) Extracts of *Sideritis scardica* as triple monoamine reuptake inhibitors. J Neural Transm 119:1477–1482

58. Dimpfel W (2013) Pharmacological classification of herbal extracts by means of comparison to spectral EEG signatures induced by synthetic drugs in the freely moving rat. J Ethnopharmacol 149:583–589

59. Hofrichter J, Krohn M, Schumacher T et al. (2016) Sideritis spp. extracts enhance memory and learning in Alzheimer's β-amyloidosis mouse models and aged C57B1/6 mice. J Alzheimer Dis 53:967–980

60. Hanlidou E, Karousou R, Kleftoyanni V et al. (2004) The herbal market of Thessaloniki (N.Greece) and its relation to the ethnobotanical tradition. J Ethnopharmacol 91:281–299

61. Dordević S, Blagojević S, Sekulović D et al. (1993) The analysis of mineral content in active components and the preparation of phytopreparations for anemia prevention. Arh Farm 43:225–231

62. Ivancheva S, Stantcheva B (2000) Ethnobotanical inventory of medicinal plants in Bulgaria. J Ethnopharmacol 69:165–172

63. http://www.theplantlist.org/tpl/record/kew-191627 [27] Datenbank The Plant List 2010 – Eintrag zu *Sideritis scardica* Griseb

64. Österreichisches Lebensmittelbuch,IV. Auflage, Codex Kapitel B 31, Tee und teeähnliche Erzeugnisse, BMGFJ-75210/0012-1V/B/7/2007 v. 20.08.2007

65. http://www.ema.europa.eu/docs/en_GB/document_library/Herbal_-_Herbal_monograph/2016/10/WC500215568.pdf [28] EMA/HMPC/39453/2015 Committee on Herbal Medicinal Products (HMPC)

66. Feistel B, Appel K (2013) Extrakte aus Griechischem Bergtee hemmen die Wiederaufnahme von Neurotransmittern. Z Phytother S01:34–P24

67. Jeremic I, Petrovic D, Zivkovic M et al. (2015) *Sideritis scardica* extract prevents bone loss in ovariectomized rats. Ann Rheum Diss 74:921

68. Heiner F, Feistel B, Wink M (2015) *Sideritis scardica* extracts inhibit the aggregation of α-synuclein and β-amyloid peptides in Caenorhabditis elegans used as a model for neurodegenerative diseases. Planta Med 81: PW_127

69. Heiner F, Feistel B, Wink M (2016) Neuroprotektive Wirkung von *Sideritis scardica*-Extrakten auf Caenorhabditis elegans, einem Modellorganismus für neurodegenerative Erkrankungen. Z Phytother 37:V22

70. Behrendt I, Schneider I, Schuchardt JP et al. (2016) Effect of an herbal extract of *Sideritis scardica* and B-vitamins on cognitive performance under stress: A pilot study. Int J Phytomed 8:95–103

71. Dimpfel W, Schombert L, Biller A (2016) Psychophysiological effects of Sideritis and Bacopa extract and three combinations thereof - A quantitative EEG study in subjects suffering from Mild Cognitive Impairment (MCI). Adv Alzheimer Dis 5:1–22

72. Dimpfel W, Feistel B, Schombert L (2016) Opposite neurophysiological findings induced by *Sideritis scardica* and *Sideritis euboa* extract in the rat. J Behav Brain Sci 6:448–461

73. Dimpfel W, Schombert L, Feistel B (2016) Ex vivo characterization of the action of Sideritis extract using electrical activity in the rat hippocampus slice preparation. Pharmacol Pharm 7:407–416

74. Petreska J, Stefova M (2012) Assay of urinary excretion of polyphenols after ingestion of a cup of mountain tea (*Sideritis scardica*) measured by HPLC- DAD-ESI-MS/MS. J Agr Food Chem 61:10488–10497

75. Dimpfel W, Biller A, Suliman S et al. (2016) Psychophysiological effects of a combination of Sideritis and Bacopa extract (memoLoges®) in 32 subjects suffering from

mild cognitive impairment. A double-blind, randomized, placebo-controlled, 2-armed study with parallel design. Adv Alzheimer Dis 5:103–125
76. http://www.ema.europa.eu/docs/en_GB/document_library/Herbal_-_List_entry/2015/07/WC500190614.pdf [29] EMA/HMPC/150543/2015, Committee on Herbal Medicinal Products (HMPC) European Union list entry on *Sideritis scardica* Griseb., herba (draft)

Copyright

Lizenzausgabe mit freundlicher Genehmigung des Springer Medizin Verlags GmbH, Berlin, Heidelberg, New York
Wissenschaftliche Verlagsgesellschaft Stuttgart, Birkenwaldstraße 44, 70191 Stuttgart

Datenstand

09.02.2018

Diese Seite ist hier zu Hause:
http://www.drugbase.de/nc/de/datenbanken/hagers-enzyklopaedie/artikel.html?tx_crondavdbhager_pi[uid]=87232&tx_solr[filter][0]=owndb%3Aall

Links auf dieser Seite:

1. http://www.drugbase.de/de/datenbanken/hagers-enzyklopaedie/gattungartdroge.html?tx_crondavdbhager_pi%5Bcategory%5D=20286&cHash=f4e45519543fd6b7c3d5cde0948d7009
2. http://www.drugbase.de/de/datenbanken/hagers-enzyklopaedie/gattungartdroge.html?tx_crondavdbhager_pi%5Bcategory%5D=53786&cHash=3681bebc72f14db52fc16ade493852e9
3. http://www.drugbase.de/de/datenbanken/hagers-enzyklopaedie/gattungartdroge.html?tx_crondavdbhager_pi%5Bcategory%5D=53787&cHash=4e3f2b9926fd08a88f5c8d68756d32ac
4. http://www.drugbase.de/de/datenbanken/hagers-enzyklopaedie/artikel.html?tx_crondavdbhager_pi%5Buid%5D=87231&cHash=12b4863b2d6bcec62a9023c12c243f47
5. http://www.drugbase.de/de/datenbanken/hagers-enzyklopaedie/artikel.html?tx_crondavdbhager_pi%5Buid%5D=87232&cHash=ccf3ce581c383768e589295b094a8083
6. http://www.drugbase.de/de/datenbanken/hagers-enzyklopaedie/artikel.html?tx_crondavdbhager_pi%5Buid%5D=87233&cHash=51071618950ffd71b801909b6233f520
7. http://www.drugbase.de/#lit_20
8. http://www.drugbase.de/#lit_45
9. http://www.drugbase.de/#lit_5
10. http://www.drugbase.de/#lit_8
11. http://www.drugbase.de/#lit_21
12. http://www.drugbase.de/#lit_22
13. http://www.drugbase.de/#lit_63
14. http://www.drugbase.de/#lit_23
15. http://www.drugbase.de/#lit_24
16. http://www.drugbase.de/#lit_26
17. http://www.drugbase.de/#lit_15
18. http://www.drugbase.de/#lit_27
19. http://www.drugbase.de/#lit_30
20. http://www.drugbase.de/#lit_31
21. http://www.drugbase.de/#lit_25
22. http://www.drugbase.de/#lit_32
23. http://www.drugbase.de/#lit_33
24. http://www.drugbase.de/#lit_11
25. http://www.drugbase.de/#lit_42
26. http://www.drugbase.de/#lit_44
27. http://www.theplantlist.org/tpl/record/kew-191627
28. http://www.ema.europa.eu/docs/en_GB/document_library/Herbal_-_Herbal_monograph/2016/10/WC500215568.pdf
29. http://www.ema.europa.eu/docs/en_GB/document_library/Herbal_-_List_entry/2015/07/WC500190614.pdf
