# Supplementary material for: Sideritis scardica extracts inhibit aggregation and toxicity of amyloid-β in Caenorhabditis elegans used as a model for Alzheimer’s disease
Source: PeerJ. 2018 Apr 30;6:e4683. doi: 10.7717/peerj.4683 (PMC5933321; doi:10.7717/peerj.4683)
Supplement: Supplemental Information 3 [file peerj-06-4683-s006.pdf]

Sideritidis scardicae herba [11] [1], [20] [2], [45] [3]

Verfasser

F. Heiner, B. Feistel, J. Reichling

Übersicht

S [4] > Sideritis [5] > Sideritis scardica GRISEB. [6] > **Sideritidis scardicae herba 11, 20, 45** [7]

Gliederung

G Sideritis [8]
A Sideritis scardica GRISEB. [9]
D **Sideritidis scardicae herba [11]** [10], **[20]** [2], **[45]** [3]

Synonyme

Herba Siteritis, Siteritis herba

Definition der Droge

Die zur Blütezeit gesammelten oder geernteten, getrockneten, oberirdischen Teile der Pflanze. Geruch: Aromatisch [11] [1], [45] [3], [46] [11], [64] [12].

Charakteristik

Stammpflanzen: Sideritis scardica GRISEB.

Herkunft: Balkan-Halbinsel; Sammlung aus Wildbeständen sowie aus dem Anbau [20] [2], [47] [13].

Pharmakognosie & Inhaltsstoffe

Inhaltsstoffe: *Flavonol-7-O-diglucoside*. Isoscutellarein-7-*O*-allosyl(1→2)glucosid, Hypolaetin-7-*O*-allosyl(1→2)glucosid, Luteolin-7-*O*-allosyl(1→2)glucosid; 3'-*O*-Methylhypolaetin-7-*O*-allosyl(1→2)glucosid, 4'-*O*-Methylisoscutellarein-7-*O*-allosyl (1→2)glucosid. *Acetylierte Flavonol-7-O-glucoside*. Hypolaetin-7-O-[6'''-*O*-acetyl] -allosyl(1→2)glucosid, Apigenin-7-*O*-[6'''-*O*-acetyl]-allosyl(1→2)glucosid, Isoscu-tellarein-7-*O*-[6'''-*O*-acetyl]-allosyl(1→2)glucoside u. a. *Hydroxyzimtsäure-Derivate*. 3-Caffeoylchinasäure, 5-Caffeoylchinasäure, Ferulasäure, Chlorogensäure, Kaffeesäure, *p*-Cumarsäure. *Phenylethanoid-Glykoside*. Echinacosid, Forsythosid, Verbascosid, Alyssonosid, Leucoseptosid [15] [14], [17] [15], [18] [16]. *Ätherisches Öl*. Im ätherischen Öl überwiegen die Monoterpene, wie z. B. α-Pinen (bis zu 50 %), β-Pinen, Carvacrol, Myrcen, 1,8-Cineol, Terpinen-4-ol u. a.; weiter findet man Sesquiterpene wie z. B. α-Cadinol (bis zu 20 %), β-Caryophyllen, Caryophyllenoxid, Germacren D, δ-Cadinen, Nerolidol, Viridiflorol u. a. [39] [17], [41] [18]. *Diterpene*. Ent-Kauren-Typ, wie z. B. Siderol (Hauptsubstanz), Isolinearol, Sideridiol, Sideroxol, Epoxysiderol, Eubol, 18-Acetoxy-Leucanthol und (E)-Ferruginolacetat [15] [14], [41] [18]. *Triterpene*. Squalen, β-Amyrin, α-Amyrin, Ursolsäure, Oleanolsäure [39] [17]. *Iridoide*. Ajugol und Ajugosid [47] [13]. Die in der Droge nachgewiesenen Mengen an sekundären Inhaltsstoffen variieren entsprechend der Witterung, den vorherrschenden Bodenverhältnissen und der jeweiligen Jahreszeit [15] [14], [39] [17], [41] [18].

Lagerung, Stabilität, Verwendung, u. a.

Gesetzliche Bestimmungen: European Union herbal monograph "Sideritis herba" vom Commitee on Herbal Medicinal Products (HMPC) mit Sitz bei der EMA (European Medicines Agency) [65] [19].

Pharmakologie

Wirkungen: Vorklinische Pharmakologie In der Vergangenheit wurden verschiedene *in vitro*-Studien (biochemische Modelle) sowie *in vivo*-Studien (Tiermodelle) durchgeführt, mit der Zielsetzung, die volkstümlichen Anwendungen der Droge [11] [1], [15] [14] wissenschaftlich zu belegen. Biochemische Modelle *Antimikrobielle Wirkung*. Abhängig vom Extraktionsmittel und der Extraktionsmethode zeigten die Extrakte *in vitro* eine antimikrobielle Aktivität gegen verschiedene Gram-positive und Gram-negative Bakterien, wie z. B. gegen Staphylococcus aureus, Staphylococcus epidermidis, Streptococcus pyogenes, Corynebacterium pseudotuberculosis, Haemophilus sp., Escherichia coli, Klebsiella pneumoniae, Pseudomonas aeruginosa. Die gemessenen minimalen Hemm-Konzentrationen (MHK-Werte) umfassten einen Bereich von 0,04 mg/mL bis zu >2 mg/mL [40] [20], [41] [18], [48] [21] - [50] [22]. *Antioxidative Wirkung*. In verschiedenen *in vitro*-Assays zeigte sich, dass vor allem die stark lipophilen Extrakte aus *Sideritis scardica* eine antioxidative Wirkung besitzen. So glich die Radikalfänger-Kapazität eines Butanol-Extraktes im DPPH-Assay in etwa der der Rosmarinsäure (94,4 bzw. 94,5 %). Diese entsprach einem IC_{50}-Wert von 5,7 µg/mL. Ethanol-Extrakte hingegen zeigten Werte von bis zu 8,9 mg/mL. Der TEAC-Wert (Trolox-Äquivalent) von einem Infus aus dem Griechischen Bergtee betrug 0,86 mM. Weiterhin wurde nachgewiesen, dass ein 70%iger methanolischer Extrakt trotz geringerem Phenolgehalt und geringerer antioxidativer Kapazität einem Camellia sinensis-Extrakt bezüglich der antioxidativen Wirkung in HepG2-Zellen ebenbürtig war. Zudem können die Extrakte zur Stabilisierung von Pflanzenölen beitragen [17] [15], [33] [23], [47] [13], [50] [22] - [54] [24]. *Zytotoxische und Anti-Glioma-Wirkung*. Es wurden dosisabhängige, zytotoxische *in vitro*-Wirkungen gegen die Zelllinien HeLa, B16 und FemX und HL-60 beschrieben; vor allem Diethylether- und Ethylacetat-Extrakte waren hier aktiv. Auf humane PBMC hatte kein getesteter Auszug eine toxische Wirkung. Zudem konnte nachgewiesen werden, dass S. scardica-Extrakte die Produktion von reaktiven Sauerstoffspezies in Gliomazellen und primären Astrozyten von Ratten erhöhen; auch Zellzyklus-Arrest und Autophagie gehören zu den Wirkmechanismen. Diese werden den enthaltenen Flavonoiden Apigenin und Luteolin bzw. ihren 7-*O*-β-Glykosiden zugeschrieben [51] [25], [52] [26], [55] [27], [56] [28]. *Antidepressive Wirkung*. Wässrige und alkoholische Extrakte hemmten in Rattenhirn-Synaptosomen die Wiederaufnahme von Serotonin, Noradrenalin und Dopamin durch Inhibition der jeweiligen Neurotransmitter-Transporter mit EC_{50}-Werten von 30 bis 40 µg/mL. In humanen JAR-Zellen inhibierten sie den Serotonintransporter (reiner Methanol-Auszug: EC_{50}=1,4 µg/mL). Ein 70%iger ethanolischer Extrakt war zudem in der Lage, die Wirkung des SSRIs Fluvoxamin zu verstärken [57] [29]. In einem weiteren Versuch mit Rattenhirn-Synaptosomen und Auszügen mit Wasser, 20 %, 40 % und 60 % Ethanol (*V*/*V*) zeigte sich, dass die stärkste Wiederaufnahmehemmung bei Noradrenalin, gefolgt von Dopamin und Serotonin stattfindet. Bei allen Neurotransmittern sanken die EC_{50}-Werte mit steigendem Anteil von Ethanol im Auszugsmittel. Der effektivste Extrakt war der 60%ige ethanolische mit einem EC_{50}-Wert von ca. 15 µg/mL für Noradrenalin und Dopamin und 60 µg/mL für Serotonin. Dieser lipophilste der getesteten Extrakte zeigte auch die stärkste Anreicherung von Flavonoiden (ca. 4 %) bzw. Polyphenolen (ca. 7 %) [66] [30]. Tiermodelle *Antiinflammatorische Wirkung*. *In vivo* erreichten Ether- und *n*-Butanol-Fraktionen eines 70%igen Ethanol-Extraktes in Dosen von 200 bzw. 100 mg/kg den gleichen Effekt wie 4 mg/kg Indometacin (Carrageenan-induziertes Rattenpfotenödem) [48] [21], [52] [26]. *Gastroprotektive Wirkung*. Verschiedene Fraktionen eines 70%igen Ethanol-Extraktes zeigten *in vivo* eine dosisabhängige, mit Ranitidin vergleichbare Wirkung, wobei die *n*-Butanol-Fraktion die wirksamste und sogar signifikant effektiver als Ranitidin war (Ethanol-induzierter Stressulcus in Ratten) [52] [26]. *Osteoprotektive Wirkung*. Weibliche, ovariektomierte Ratten, welche mit einem ethanolischen Auszug behandelt wurden, zeigten eine höhere Knochendichte, eine höhere mechanische Belastbarkeit der Knochen und verringerte kardiovaskuläre metabolische Risikofaktoren, die mit der Menopause assoziiert sind [67] [31]. *Antidepressive und psychostimulierende Wirkung*. Im Vergleich ähnelten die EEG-Frequenzmuster von mit *S. scardica*-Extrakt (20 %, *V*/*V* ethanolisch) behandelten Ratten denen von mit Methylphenidat (einem Psychostimulanz), Tramadol bzw. Paroxetin (beide haben antidepressive Eigenschaften) und mit *Ginkgo biloba*-Extrakt behandelten Ratten. Dabei zeigte *Sideritis euboa*, eine andere Sideritis-Art, trotz ähnlicher Neurotransmitter-Wiederaufnahme Henmmungen bei Serotonin und Dopamin *in vitro*, sehr gegensätzliche Frequenzmuster und wird deswegen nicht als (Gedächtnis-) stimulierend eingeschätzt [58] [32], [72] [33]. Bei der positiven Wirkung des 20%igen (*V*/*V*) ethanolischen *S. scardica*-Extraktes auf die Gehirnfunktion ist wahrscheinlich auch eine Modulation der AMPA-abhängigen Neurotransmission beteiligt, da eine *S. scardica*-induzierte elektrische Aktivität im Hippocampus von Ratten von einem Antagonisten dieses Glutamat-Rezeptors verhindert werden konnte. Andere Glutamat-Rezeptoren waren nicht beteiligt [73] [34]. *Gedächtnisleistungssteigernde und neuroprotektive Wirkung*. Im Morris-Wasserlabyrinth fanden sowohl transgene Alzheimer-Mäuse, als auch gealterte, nicht-transgene Mäuse, die mit einem 20%igen (*V*/*V*) Ethanol-Extrakt behandelt wurden, signifikant schneller die rettende Plattform als die jeweiligen Kontrolltiere. Bei den nicht-transgenen Tieren ließ sich dieses Ergebnis zu jedem herangezogenen Zeitpunkt während des Langzeitversuchs (600 Tage) beobachten. Dies lässt auf eine Verbesserung des Kurzzeitgedächtnisses und der Kognition der Tiere in jedem Alter schließen. Weiterhin konnte eine gesteigerte phagozytische Aktivität der Microglia und eine erhöhte Expression der α-Sekretase ADAM10 ermittelt werden. Beides kann die Last am für Alzheimer

charakteristischen β-Amyloid-Peptid verringern. Tatsächlich reduzierte die Behandlung mit *Sideritis scardica*-Extrakt die β-Amyloid-Plaques in den Maushirnen sowohl in Zahl, als auch in Größe (je ca. -30 %). Auch die Menge an löslichem Aβ verringerte sich um mehr als die Hälfte [59] [35]. Im *Caenorhabditis elegans*-Modell konnten ebenfalls Belege für eine Wirksamkeit gegen die Charakteristika der Alzheimer- als auch der Parkinsonerkrankung gefunden werden. So reduzierte sich auch in diesem Modellorganismus die Zahl der β-Amyloid-Plaques. Aber auch eine verringerte Toxizität der Aβ-Peptide bzw. eine dadurch verringerte Neurodegeneration und damit einhergehende Besserung einer kognitiven Störung konnte nachgewiesen werden. Weiterhin verminderten die ethanolischen Auszüge aus *S. scardica* die Aggregation von α-Synuclein und wirkten neuroprotektiv auf dopaminerge Neuronen [68] [36], [69] [37]. Klinische Wirksamkeit *Kognitive leistungssteigernde Wirkung.* In einer Pilotstudie mit 64 gesunden Probanden konnte eine Verbesserung der kognitiven Leistung unter Stressbedingungen nach der Einnahme eines Nahrungsergänzungsmittels mit 20%igem (*V*/*V*) ethanolischem *S. scardica*-Extraktes und B-Vitaminen gezeigt werden. Hierzu mussten Konzentrationstests vor und nach akuten Stressstimuli absolviert werden [70] [38]. In einer Anwendungsbeobachtung, welche 10 Probanden mit MCI (Mild Cognitive Impairment) umfasste, wurden bessere Ergebnisse in einem d2-Konzentrationstest nach Behandlung mit *Sideritis scardica*-Extrakt (20 %, *V*/*V*, Ethanol) allein und in Kombination mit *Bacopa monnieri*-Extrakt erzielt. Dabei zeigte sich während einer quantitativen EEG-Messung vor allem Aktivität in fronto-temporalen Hirnbereichen [71] [39]. In einer randomisierten, Placebo-kontrollierten, zweiarmigen Doppelblindstudie mit Paralleldesign konnte anhand von 32 Probanden mit MCI festgestellt werden, dass nach der Einnahme eines Kombinationspräparates aus *S. scardica* und *B. monnieri* verschiedene Konzentrations- und Gedächtnistests besser absolviert wurden. Dabei verstärkte das Kombinationspräparat während der mentalen Forderung signifikant die Aktivität von Beta-Wellen im Gehirn, während es in Erholungsphasen, wie z. B. durch das Antidementivum Rivastigmin hervorgerufen, die Aktivität von Delta- und Theta-Wellen dämpfte [75] [40]. *Bioverfügbarkeit.* In einer Studie an 10 Probanden (HPLC-MS-Messung von Urinproben 24 h nach Einnahme eines Dekokts) wurden nur etwa 5 % der aufgenommenen Polyphenole im Urin als Metabolite gefunden. Dabei handelte es sich um sulfatierte, glucuronidierte oder methylierte Flavonoide, Chinasäure- und Phenylpropanoid-Derivate [74] [41].

Anwendungsgebiete

Traditionelle Anwendung (traditional use) auf Grund langjähriger Erfahrung: a) zur Linderung von Husten bei Erkältung; b) zur Linderung von leichten Beschwerden des Magen-Darm-Traktes (gastrointestinale Beschwerden) [65] [19].

Dosierung & Art der Anwendung

Teeaufguss. 2 bis 4 g zerkleinerte Droge auf 150 bis 200 ml Wasser, zum Einnehmen 2 bis 3x tgl.; max. Tagesdosis: 12 g Droge [65] [19].

Volkstümliche Anwendungen & andere Anwendungsgebiete

Im Herkunftsgebiet hat die Droge den Ruf, antimikrobielle, antiinflammatorische, antirheumatische und gastroprotektive Eigenschaften zu besitzen. Auch eine lebensverlängernde Wirkung wird dem Kraut nachgesagt. Genutzt wird es volksmedizinisch als Schleimlöser bei Bronchitis und bronchialem Asthma, gegen Erkältungen, Halsschmerzen und Husten, zur Behandlung von Entzündungen jeglicher Art, bei Magen-Darm-Beschwerden, bei Lungenemphysemen und zur Prävention einer Anämie. Auch bei Rhinitis, zur Entgiftung des Körpers, als Diaphoretikum, bei Prostatahyperplasie, zur Wundheilungsförderung und bei Lippenherpes werden Zubereitungen vom Griechischen Bergtee angewendet. Auf chronische Nierenerkrankungen soll *Sideritis scardica* einen positiven Einfluss durch einen diuretischen Effekt haben. In Bulgarien wurde hierzu eine Anwendungsbeobachtung mit dem Teeaufguss durchgeführt. Alkoholische Tinkturen werden sowohl innerlich als auch äußerlich, z. B. als Antiseptikum nach dem Zähneziehen oder bei Infektionen des Mundraums genutzt. Die beanspruchten Wirkungen und Anwendungsgebiete sind derzeit nicht ausreichend belegt. Neben medizinischen werden auch kosmetische Anwendungen beschrieben [11] [1], [15] [14], [60] [42] - [62] [43]. Als Infus heiß oder kalt über den Tag verteilt getrunken (1 bis 2 L). Aufgegossen werden häufig 3 bis 5 blühende Stängel mit 1 L kochendem Wasser für 20 bis 30 min. Oft werden andere endemische Pflanzen hinzugefügt, um einen gemischten Kräutertee zu erhalten. Auch die Zugabe von Zitronensaft und Honig oder Zimt zur Herstellung eines Erfrischungsgetränkes ist üblich [11] [1], [15] [14], [17] [15], [46] [11]. Eine Tinktur (1:5) aus Ethanol 60 % ist kommerziell erhältlich und wird mit einer Dosis von 3x tgl. 1,6 mL angegeben. Dabei entspricht die Gesamtdosis von 4,8 mL einem Drogenäquivalent von 1,2 g Droge (Sideritidis scardicae herba) gemäß Herstellerangaben. Ein kommerzieller, Tee-analoger Flüssigextrakt zur Nahrungsergänzung wird mit einer Verzehrempfehlung von 10 mL pro Tag angegeben. Ein hydroethanolisch hergestellter Trockenextrakt (Auszugsmittel Ethanol 20 %, *V*/*V*) ist als Nahrungsergänzungsmittel zur kognitiven Leistungssteigerung in einem Kombinationspräparat mit 760 mg Tagesdosis seit 2016 im deutschen Markt verfügbar.

Toxikologie

Unbedenklichkeit. Der Einsatz des Griechischen Bergtees lässt sich seit Mitte des 20. Jahrhunderts, darunter in Österreich und Bulgarien, nachvollziehen [64] [12].

Acute Toxizität:

Tier. Ein 20%iger (*V*/*V*) ethanolischer Extrakt rief - verabreicht als orale Einzeldosis von 2000 mg/kg über eine Dauer von 14 Tagen - keine histopathologischen Abnormitäten oder Zeichen von Toxizität in Sprague Dawley-Ratten hervor (nach OECD-Richtlinie zur Prüfung von Chemikalien, Nr. 423, Sektion 4). Der getestete Extrakt wurde der Kategorie (GHS) zugewiesen, welche eine orale LD_{50} von 2000 bis 5000 mg/kg Körpergewicht abdeckt [65] [19].

Chronische Toxizität:

Tier. Derselbe Extrakt (20 %, *V*/*V* EtOH) wurde in insgesamt 60 Sprague Dawley-Ratten über 28 Tage mit einer 14-tägigen Erholungsphase auf eine potentielle Toxizität bei Mehrfachgabe hin untersucht (nach OECD-Richtlinie, Nr. 407). Dabei erhielten die Tiere oral täglich entweder destilliertes Wasser (Kontrolle), 250, 500 oder 1000 mg/kg des Extraktes über 4 Wochen. Alle Ratten überlebten und zeigten weder ein verändertes Körper- oder Organgewicht, auch keine schlechtere Futteraufnahme noch irgendwelche pathologischen Zeichen von Toxizität über den gesamten Versuchszeitraum inkl. Erholungsphase. Ebenfalls unbeeinflusst blieben Hör- und Sehvermögen sowie Propriozeption (Tiefensensibilität; u.a. Wahrnehmung von Körperlage und -bewegung im Raum). Hämatologische und biochemische Parameter (Calcium, Natrium, Chlorid) wurden nur marginal beeinflusst. Das NOAEL des geprüften *Sideritis scardica*-Extraktes wurde deswegen von den Autoren auf 1000 mg/kg Körpergewicht festgelegt [65] [19].

Mutagen: Konform mit GLP- und ICH-Richtlinien sowie dem Bracketing/Matrixing-Modell (EMEA/HMPC/67644/2009) wurden vier *S. scardica*-Extrakte unterschiedlicher Polarität (Auszugsmittel Wasser, Ethanol 20 und 50 % (*V*/*V*) sowie *n*-Heptan) im Ames-Test auf potentiell mutagene Eigenschaften hin untersucht. Diese Auszüge decken die gesamte Bandbreite an phytochemischen Inhaltsstoffen der Droge von polar bis unpolar ab. Zur Testung wurden je 5 Stämme *Salmonella typhimurium* mit und ohne metabolischer Aktivierung herangezogen. Keiner der getesteten Extrakte zeigte in den getesteten Konzentrationen (je bis zu 5000 µ/Platte) mit oder ohne metabolischer Aktivierung eine gegenüber der Kontrolle erhöhte Zahl an Revertanten-Kolonien. *Sideritis scardica* wurde als nicht mutagen eingestuft [65] [19]. Die sehr gute Datenlage zu *Sideritis scardica* führte über die Evaluierung im HMPC zu einem vorläufigen "List entry" beim HMPC, was nur für besonders sichere traditionelle Pflanzenzubereitungen ausgesprochen wird [76] [44].

Literatur

1. Scheen AC, Bendiksby, M, Ryding O et al. (2010) Molecular phylogenetics, character evolution, and suprageneric classification of Lamioideae (Lamiaceae). Ann Missouri Bot Gard 97:191 217
2. Salmaki Y, Zarre S, Ryding O et al. (2013) Molecular phylogeny of tribe Stachydeae (Lamiaceae subfamily Lamioideae). Mol Phylogenet Evol 69:535 551
3. Huynh KL (1972) Le pollen et la systématique du genre Sideritis L. (Labiatae). Bull Museum Natl Hist Nat (Paris) 45:1 28
4. Huber-Morath A (1982) Sideritis. In: Peter Hadland Davis (Eds.): Flora of Turkey and the East Aegean Islands. Vol. 7 (Orobanchaceae to Rubiaceae). Edinburgh University Press, S. 178 199
5. Papanikolaou K, Kokkini S (1982) A taxonomic revision of Sideritis L. section Empedoclia (Rafin.) Benntham (Labiatae) in Greece. In: Margaris, N., Koedam, A., Vokou, D. (Eds.), Aromatic Plants: Basic and Applied Aspects. Artinus Nijhov Publ., The Hague, S. 101 128
6. Pérez de Paz P, Negrín-Sosa L (1992) Revisión taxonómica de Sideritis L. Subgénero Marrubiastrum (Moench) Mend.-Heur. Phanerogamarum Monographiae, Tomus XX. J. Cramer, Berlin
7. Obón de Castro C, Rivera-Núñez D (1994) A taxonomic revision of the section Sideritis (genus Sideritis) (Labiatae). Phanerogamarum Monographiae Tomus XXI. J. Cramer, Berlin
8. Cantino PD, Abu-Asab MS (1994) Systematic implications of pollen morphology in subfamilies Lamioideae and Pogostemonoideae (Labiatae). Ann Missouri Bot Gard 81:653 686
9. Morales R (2010) Sideritis. In: Castroviejo S, Morales R, Quintana A, et al. (Eds.), Flora Iberica XII. Plantas vasculares de la Península Ibérica e Islas Baleares. Real Jardín Botánico, CSIC, Madrid, S 1 56

10. Bendiksby M, Lisbeth T, Scheen AC et al. (2011) An updated phylogeny and classification of Lamiaceae subfamily Lamioideae. Taxon 60:471 484
11. González-Burgos E, Carretero ME, Gómez-Serranillo MP (2011) Sideritis spp.: uses, chemical composition and pharmacological activities - a review. J Ethno pharmacol 135:209 225
12. Güvenç A, Duman H (2010) Morphological and anatomical studies of annual taxa of Sideritis L. (Lamiaceae), with notes on chorology in Turkey. Turk J Bot 34:83 104
13. Davis PH (1982) Flora of Turkey and the East Aegean Islands. Edinburgh University Press, Edinburgh, S. 7
14. Davis PH, Mill RR, Kit Tan (1988) Flora of Turkey and the East Aegean Islands. Edinburgh University Press, Edinburgh, S. 10
15. Todorova M, Trendafilova A (2014) *Sideritis scardica* Griseb., an endemic species of Balkan peninsula: Traditional uses, cultivation, chemical composition, biologicalactivity. Review. J Ethnopharmacol 152:256 265
16. Fraga BM (2012) Phytochemistry and chemotaxonomy of Sideritis species from the Mediterranean region. Phytochem 76:7 24
17. Petreska J, Stefkov G, Kulevanova S et al. (2011a) Phenolic compounds of mountain tea from the Balkans: LC/DAD/ESI/MSn profile and content. Nat Prod Commun 6:21 30
18. Petreska J, Stefova M, Ferreres F et al. (2011b) Potential bioactive phenolics of Macedonian Sideritis species used for medicinal "Mountain Tea". Food Chem 125:13 20
19. Bojovic D, Jankovic S, Potpara Z et al. (2011) Summary of the phytochemical research performed to date on Sideritis species. Ser J Exp Clin Res 12:109 122
20. Heywood V (1972) Sideritis L. In: Tutin T, Heywood V, Burges N et al. (Eds.), Flora Europaea, 3. Cambridge University Press, Cambridge, S. 134 138
21. Lim TK (2014) Edible Medicinal and Non Medicinal Plants. Volume 8, Flowers. Dordrecht: Springer Science + Business Media
22. Halácsy E (1902) Conspectus Florae Graecae. G.Engelmann, Lipsiae, Vol. II, 497–502
23. Feistel B (2013) Griechisches Eisenkraut - Mental fit mit Griechischem Bergtee. Vitalstoffe 3:34–37
24. Assenov I, Ganchev G (1978) Morphological characteristics of *Sideritis scardica* Griseb. Pharmacia 2:29–32
25. Evstatieva L, Bakalova K, Protich N (1990) Ecological and biological peculiarities of *Sideritis scardica* Griseb. Plant Sci. 27:77–80 (bulgar.)
26. Evstatieva L (2011) *Sideritis scardica* Griseb. In: Peev D (Ed.), Red Data Book of the Republic of Bulgaria, Joint Edition of the Bulgarian Academy of Sciences & Ministry of Environment and Water of Bulgaria, Digital Edition, Vol. 1, http:// e-ecodb.bas.bg/rdb/en/
27. Baden C (1991) Sideritis L. In: Strid A, Tan K (Eds.), The Mountain Flora of Greece, 2. Edinburgh University Press, Edinburgh, S. 84–91
28. Duman H (2000) Sideritis L. In: Güner A, Ozhatay N, Ekim T, Basher KHC (Eds.), Flora of Turkey and the East Aegean Islands, 11. Edinburgh University Press, Edinburgh, S. 201–204
29. Yordanova M, Apostolova I (2000) Estimation of the status of representative populations of *Sideritis scardica* Griseb. in the Rhodopi Mts. Phytol Balc 6:4357
30. Petrova A, Vladimirov V (2009) Red list of Bulgarian vascular plants. Phytol Balc 15:63–94
31. Skrubis G (1984) The Greek mountain tea. In: Spice, medicinals, aromatics. Department of aromatic plants, agriculture research center of northern Greece Thessaloniki - Greece. Acta Horticultura 144:25–29
32. Evstatieva L (2002) Variation in the flavonoids and tannins content of S. scardica Griseb. Annual of Sofia University "Kliment Ohridski" 90. Faculty of Biology, Sofia, S. 99–105
33. Karapandzova M, Qazimi B, Stefkov G et al. (2013) Chemical characterization, mineral content and radical scavenging activity of *Sideritis scardica* and *S. raeseri* from R. Macedonia and R. Albania. Nat Prod Commun 8:639–644
34. Kokkalou E (1987) Constituents entrainables à la vapeur d'eau de *Sideritis scardica* Griseb subsp. scardica. Plant Med Phytother 21:262–266
35. Baser KHC, Kirimer N, Tümen G (1997) Essential oil of *Sideritis scardica* Griseb subsp. scardica. J Essent Oil Res 9:205–207
36. Kostadinova E, Nikolova D, Alipieva K et al. (2007) Chemical constituents of the essential oils of *Sideritis scardica* Griseb. and *Sideritis raeseri* Boiss and Heldr. from Bulgaria and Macedonia. Nat Prod Res 21:319–
37. Todorova M, Christov R, Evstatieva L (2000) Essential oil composition of three Sideritis species from Bulgaria. J Essent Oil Res 12:418–420
38. Trendafilova AB, Todorova MN, Evstatieva LN et al. (2013) Variability in the essential-oil composition of *Sideritis scardica* Griseb. from native bulgarian populations. Chem Biodivers 10:484–492
39. Qazimi B, Karapandzova M, Stefkov G et al. (2010) Chemical composition of ultrasonic assisted n-hexane extracts of *Sideritis scardica* Grieseb. and *Sideritis raeseri* Boiss. & Heldr. (Lamiaceae) from Macedonia and Albania. Maced. Pharmaceut Bull 56:45–56
40. Kostadinova E, Alipieva K, Stefova M et al. (2008) Influence of cultivation on the chemical composition and antimicrobial activity of Sideritis spp. Pharmacogn Mag 4:102 –106
41. Tadić V, Bojović D, Arsić I et al. (2012a) Chemical and antimicrobial evaluation of supercritical and conventional *Sideritis scardica* Griseb. (Lamiaceae) extracts. Molecules 17:2683–2703
42. Taskova R, Mitova M, Anchev M et al. (1997) Iridoids, flavonoids and terpenoids as taxonomic markers in Lamiaceae, Scrophulariaceae and Rubiaceae. Bocconea 5:631–636
43. Venturella P, Bellino A (1979) Isolation and partial synthesis of ent-18-acetoxykaur-16-ene-3β,7α,15β-triol from *Sideritis scardica*. Phytochem 1:1571–1572
44. Žarković B, Djordjević S, Blagojević S (1993) Comparison of the mineral composition of the plant *Sideritis scardica* Griseb., Lamiaceae and corresponding soil substrate. Arh Farm 43:233–237
45. Nikolov S (2006) Encyclopedia of the Medicinal Plants in Bulgaria. Publishing House "Trud", Sofia, S. 347–348. (bulgar.)
46. Bojchinov A (1943) Pharmacognostic study of *Sideritis scardica* Griseb. Aptek Pregled 9:151–158 (bulgar.)
47. Koleva I, Linssen JPH, van Beek TA et al. (2003) Sideritis species (Labiatae) grown in Bulgaria. J Sci Food Agric 83:809–819
48. Tadić VM, Djordjević S, Arsić I et al. (2007) Anti-inflammatory and antimicrobial activity of *Sideritis scardica* extracts. Planta Med 73:P-098
49. Proestos C, Zoumpoulakis P, Sinanoglou VJ (2013) Determination of plant bioactive compounds. Antioxidant capacity and antimicrobial screening. FMFI 2:26–
50. Mihaylova DSP, Anna L, Albert KI (2014) A study on the antioxidant and antimicrobial activities of pressurized-liquid extracts of *Clinopodium vulgare* and *Sideritis scardica*. Agro Food Ind Hi Tech 25:55–58
51. Tadić VM, Djordjević S, Arsić I et al. (2008) Cytotoxic activity and antioxidative properties of *Sideritis scardica* extracts, Planta Med 74:PA206
52. Tadić VM, Jeremic I, Dobric S et al. (2012) Anti-inflammatory, gastroprotective, and cytotoxic effects of *Sideritis scardica* extracts. Planta Med 78:415–427
53. Ivanova D, Gerova D, Chervenkov T et al. (2005) Polyphenols and antioxidant capacity of Bulgarian medicinal plants. J Ethnopharmacol 96:145–150
54. Danesi F, Saha S, Kroon PA et al. (2013) Bioactive-rich *Sideritis scardica* tea (mountain tea) is as potent as *Camellia sinensis* tea at inducing cellular antioxidant defences and preventing oxidative stress. J Sci Food Agric 93: 3558–3564
55. Tadić VM, Marković G, Jeremic et al. (2009) Antiglioma action of *Sideritis scardica* extracts, Planta Med 75:PE67 (DOI: 10.1055/s-0029-1234628).
56. Jeremić I, Tadić V, Isaković A et al. (2013) The mechanisms of in vitro cytotoxicity of mountain tea, *Sideritis scardica*, against the C6 glioma cellline. Planta Med 79:1516 –1524
57. Knörle R (2012) Extracts of *Sideritis scardica* as triple monoamine reuptake inhibitors. J Neural Transm 119:1477–1482
58. Dimpfel W (2013) Pharmacological classification of herbal extracts by means of comparison to spectral EEG signatures induced by synthetic drugs in the freely moving rat. J Ethnopharmacol 149:583–589
59. Hofrichter J, Krohn M, Schumacher T et al. (2016) Sideritis spp. extracts enhance memory and learning in Alzheimer's β-amyloidosis mouse models and aged C57B1/6 mice. J Alzheimer Dis 53:967–980
60. Hanlidou E, Karousou R, Kleftoyanni V et al. (2004) The herbal market of Thessaloniki (N.Greece) and its relation to the ethnobotanical tradition. J Ethnopharmacol 91:281–299
61. Dordević S, Blagojević S, Sekulović D et al. (1993) The analysis of mineral content in active components and the preparation of phytopreparations for anemia prevention. Arh Farm 43:225–231
62. Ivancheva S, Stantcheva B (2000) Ethnobotanical inventory of medicinal plants in Bulgaria. J Ethnopharmacol 69:165–172
63. http://www.theplantlist.org/tpl/record/kew-191627 [45] Datenbank The Plant List 2010 – Eintrag zu *Sideritis scardica* Griseb
64. Österreichisches Lebensmittelbuch,IV. Auflage, Codex Kapitel B 31, Tee und teeähnliche Erzeugnisse, BMGFJ-75210/0012-1V/B/7/2007 v. 20.08.2007
65. http://www.ema.europa.eu/docs/en_GB/document_library/Herbal_-_Herbal_monograph/2016/10/WC500215568.pdf [46] EMA/HMPC/39453/2015 Committee on Herbal Medicinal Products (HMPC)

66. Feistel B, Appel K (2013) Extrakte aus Griechischem Bergtee hemmen die Wiederaufnahme von Neurotransmittern. Z Phytother S01:34–P24
67. Jeremic I, Petrovic D, Zivkovic M et al. (2015) *Sideritis scardica* extract prevents bone loss in ovariectomized rats. Ann Rheum Diss 74:921
68. Heiner F, Feistel B, Wink M (2015) *Sideritis scardica* extracts inhibit the aggregation of α-synuclein and β-amyloid peptides in Caenorhabditis elegans used as a model for neurodegenerative diseases. Planta Med 81: PW_127
69. Heiner F, Feistel B, Wink M (2016) Neuroprotektive Wirkung von *Sideritis scardica*-Extrakten auf Caenorhabditis elegans, einem Modellorganismus für neurodegenerative Erkrankungen. Z Phytother 37:V22
70. Behrendt I, Schneider I, Schuchardt JP et al. (2016) Effect of an herbal extract of *Sideritis scardica* and B-vitamins on cognitive performance under stress: A pilot study. Int J Phytomed 8:95–103
71. Dimpfel W, Schombert L, Biller A (2016) Psychophysiological effects of Sideritis and Bacopa extract and three combinations thereof - A quantitative EEG study in subjects suffering from Mild Cognitive Impairment (MCI). Adv Alzheimer Dis 5:1–22
72. Dimpfel W, Feistel B, Schombert L (2016) Opposite neurophysiological findings induced by *Sideritis scardica* and *Sideritis euboa* extract in the rat. J Behav Brain Sci 6:448–461
73. Dimpfel W, Schombert L, Feistel B (2016) Ex vivo characterization of the action of Sideritis extract using electrical activity in the rat hippocampus slice preparation. Pharmacol Pharm 7:407–416
74. Petreska J, Stefova M (2012) Assay of urinary excretion of polyphenols after ingestion of a cup of mountain tea (*Sideritis scardica*) measured by HPLC- DAD-ESI-MS/MS. J Agr Food Chem 61:10488–10497
75. Dimpfel W, Biller A, Suliman S et al. (2016) Psychophysiological effects of a combination of Sideritis and Bacopa extract (memoLoges®) in 32 subjects suffering from mild cognitive impairment. A double-blind, randomized, placebo-controlled, 2-armed study with parallel design. Adv Alzheimer Dis 5:103–125
76. http://www.ema.europa.eu/docs/en_GB/document_library/Herbal_-_List_entry/2015/07/WC500190614.pdf [47] EMA/HMPC/150543/2015, Committee on Herbal Medicinal Products (HMPC) European Union list entry on *Sideritis scardica* Griseb., herba (draft)

Copyright

Lizenzausgabe mit freundlicher Genehmigung des Springer Medizin Verlags GmbH, Berlin, Heidelberg, New York
Wissenschaftliche Verlagsgesellschaft Stuttgart, Birkenwaldstraße 44, 70191 Stuttgart

Datenstand

09.02.2018

Diese Seite ist hier zu Hause:
http://www.drugbase.de/nc/de/datenbanken/hagers-enzyklopaedie/artikel.html?tx_crondavdbhager_pi[uid]=87233&tx_solr[filter][0]=owndb%3Aall

Links auf dieser Seite:
1. http://www.drugbase.de/#lit_11
2. http://www.drugbase.de/#lit_20
3. http://www.drugbase.de/#lit_45
4. http://www.drugbase.de/de/datenbanken/hagers-enzyklopaedie/gattungartdroge.html?tx_crondavdbhager_pi%5Bcategory%5D=20286&cHash=f4e45519543fd6b7c3d5cde0948d7009
5. http://www.drugbase.de/de/datenbanken/hagers-enzyklopaedie/gattungartdroge.html?tx_crondavdbhager_pi%5Bcategory%5D=53786&cHash=3681bebc72f14db52fc16ade493852e9
6. http://www.drugbase.de/de/datenbanken/hagers-enzyklopaedie/gattungartdroge.html?tx_crondavdbhager_pi%5Bcategory%5D=53787&cHash=4e3f2b9926fd08a88f5c8d68756d32ac
7. http://www.drugbase.de/de/datenbanken/hagers-enzyklopaedie/gattungartdroge.html?tx_crondavdbhager_pi%5Bcategory%5D=53788&cHash=86ab473f54dcf369538694796e588c72
8. http://www.drugbase.de/de/datenbanken/hagers-enzyklopaedie/artikel.html?tx_crondavdbhager_pi%5Buid%5D=87231&cHash=12b4863b2d6bcec62a9023c12c243f47
9. http://www.drugbase.de/de/datenbanken/hagers-enzyklopaedie/artikel.html?tx_crondavdbhager_pi%5Buid%5D=87232&cHash=ccf3ce581c383768e589295b094a8083
10. http://www.drugbase.de/de/datenbanken/hagers-enzyklopaedie/artikel.html?tx_crondavdbhager_pi%5Buid%5D=87233&cHash=51071618950ffd71b801909b6233f520
11. http://www.drugbase.de/#lit_46
12. http://www.drugbase.de/#lit_64
13. http://www.drugbase.de/#lit_47
14. http://www.drugbase.de/#lit_15
15. http://www.drugbase.de/#lit_17
16. http://www.drugbase.de/#lit_18
17. http://www.drugbase.de/#lit_39
18. http://www.drugbase.de/#lit_41
19. http://www.drugbase.de/#lit_65
20. http://www.drugbase.de/#lit_40
21. http://www.drugbase.de/#lit_48
22. http://www.drugbase.de/#lit_50
23. http://www.drugbase.de/#lit_33
24. http://www.drugbase.de/#lit_54
25. http://www.drugbase.de/#lit_51
26. http://www.drugbase.de/#lit_52
27. http://www.drugbase.de/#lit_55
28. http://www.drugbase.de/#lit_56
29. http://www.drugbase.de/#lit_57
30. http://www.drugbase.de/#lit_66
31. http://www.drugbase.de/#lit_67
32. http://www.drugbase.de/#lit_58
33. http://www.drugbase.de/#lit_72
34. http://www.drugbase.de/#lit_73
35. http://www.drugbase.de/#lit_59
36. http://www.drugbase.de/#lit_68
37. http://www.drugbase.de/#lit_69
38. http://www.drugbase.de/#lit_70
39. http://www.drugbase.de/#lit_71
40. http://www.drugbase.de/#lit_75
41. http://www.drugbase.de/#lit_74
42. http://www.drugbase.de/#lit_60
43. http://www.drugbase.de/#lit_62
44. http://www.drugbase.de/#lit_76

45. http://www.theplantlist.org/tpl/record/kew-191627
46. http://www.ema.europa.eu/docs/en_GB/document_library/Herbal_-_Herbal_monograph/2016/10/WC500215568.pdf
47. http://www.ema.europa.eu/docs/en_GB/document_library/Herbal_-_List_entry/2015/07/WC500190614.pdf
